# Men's health: non-communicable chronic diseases and social vulnerability**^1^**

**DOI:** 10.1590/1518-8345.0735.2756

**Published:** 2016-08-15

**Authors:** Daniele Natália Pacharone Bertolini Bidinotto, Janete Pessuto Simonetti, Silvia Cristina Mangini Bocchi

**Affiliations:** 2 MSc, Assistant Professor, Faculdade de Ciências da Saúde de Barretos Dr. Paulo Prata, Barretos, SP, Brazil.; 3 PhD, Assistant Professor, Faculdade de Medicina de Botucatu, Universidade Estadual Paulista "Julio de Mesquita Filho", Botucatu, SP, Brazil.

**Keywords:** Men's Health, Primary Health Care, Geographic Mapping

## Abstract

**Objectives:**

to evaluate the relationship between absences in scheduled appointments and the number of non-communicable chronic diseases and to investigate the relationship between spatial distribution of these diseases and social vulnerability, using geoprocessing.

**Method:**

a quantitative study of sequential mixed approach by analyzing 158 medical records of male users to relate the absences and 1250 medical records for geoprocessing

**Results:**

the higher the number of absences in the scheduled medical appointments, the less were the number of non-communicable chronic diseases and the ones listed in the International Classification of Diseases in single men. There were 21 significant geostatistically cases of glucose intolerance in the urban area. Of these, 62% lived in a region with a social vulnerability rating of Very Low, Medium 19%, 14% Low and 5% High.

**Conclusion:**

it was observed that the older the men, the greater is the number of chronic diseases and the less they miss scheduled appointments. Regarding the use of geoprocessing, we obtained a significant number of cases of glucose intolerance in urban areas, the majority classified as Very Low social vulnerability. It was possible to relate the spatial distribution of these diseases with the social vulnerability classification; however, it was not possible to perceive a relationship of them with the higher rates of social vulnerability.

## Introduction

Men reject the possibility of becoming ill, possibly because it is difficult for them to recognize their health needs. Consequently, they have a higher mortality and lower life expectancy compared to women ^(^ ^1^. 

Recognizing the context, the Ministry of Health created the National Policy for Integral Attention to Men's Health (PNAISH-in Portuguese), that has as a challenge to mobilize Brazilian men to fight and guarantee their social right to health. Geared towards those challenges, the PNAISH aligned itself to the National Primary Care Policy (PNAB - In Portuguese), with the humanization of strategies and in consonance with the principles of the Brazilian National Health System (SUS - In Portuguese), to strengthen actions and services on networks and health care^1^.

Although released in 2009, PNAISH is not yet being used in its essence, specific activities being implemented, such as the campaigns of prostate cancer prevention, in most parts of Brazil.

This context related to men´s health does not occur only in Brazil and other developing countries. A research conducted in England noted that while men are seen as "difficult to access group", little attention has been given to health policies to assess the easiness and difficulties that interfere with preventative activities with men ^(^ ^2^. As well as a research conducted in the United States showed a shortage of resources for the health of men, significantly lower when compared to those dedicated to other population segments^3^.

In addition to the health systems not prioritizing attention to health of adult men, the training of professionals who serve this population needs to be discussed and aligned to its needs. A Survey in Malaysia showed that doctors still have a fragmented view of men, not focused in terms of health, they don't encourage these men to seek health services, they have doubts about whether to serve them in a generalized way or only with a sexual focus, and few of these professionals give priority to chronic diseases^4^. 

Thus, it appears that the main reasons of the survival rate being lower among men compared to women, are related to the little investment in the operationalization of health policies for them, as well as in the training of health professionals prepared to meet specific needs related to sex.

In Singapore, men die earlier and suffer from heart disease, cerebrovascular disease, sexually transmitted diseases and cancer. Mental health issues were also significant, with men having higher suicide rate than women. These conditions result in years of productive life lost. They, more than women, have an unhealthy lifestyle that predisposes to chronic diseases ^(^ ^5^ which constitute the largest health problem in Brazil and worldwide^6^. 

To satisfy this objective, it is necessary to know the personal and socioeconomic characteristics of the male population to intervene in their health, in the scope of health promotion and prevention of injuries.

One of the ways that enables handling health data in a spatial form is geoprocessing. The use of maps has been effective to identify the most affected areas and their spatial impact, adapting the needs of health professionals in the analysis of epidemiological settings ^(^ ^7^.

In order to identify and classify the regions of the municipalities in a situation of greater or lesser vulnerability to which the population is exposed from a gradient of socioeconomic and demographic profile, the State System of Data Analysis Foundation (SEADE - In Portuguese) created the Paulista Index of Social Vulnerability (IPVS- In Portuguese). This index was developed with the intention of offering the public manager and society in general, a more detailed view of the living conditions found in the cities belonging to the State of São Paulo, with the identification and spatial localization of the areas that house the population segments more vulnerable to poverty. These regions were classified into groups ranging from very low to very high vulnerability, foreseeing the high and unclassified vulnerability of rural sectors ^(^ ^8^. 

Considering the man as a user of the primary care network health, they asked: are absences in scheduled appointments related to the number of non-communicable chronic diseases (NCCD)? There is a relationship of Social Vulnerability (SV) with the number of NCCD?

The presumed hypotheses is: there is a relationship between the percentage of the absence of men in the scheduled visits and the number of NCCD, as well as between the spatial distribution of NCCDs and the spatial distribution of SV .

The objectives of this research were to evaluate the relationship between absences in scheduled appointments and the number of NCCD and to investigate the relationship between the spatial distribution of these diseases and SV , using geoprocessing.

## Method

The inclusion criteria of these units were: the use of the Municipal System Health Information program (SIMIS in Portuguese), 2011 being the last year of its use, since from 2012 on a new system was implemented in stages; have completed at least three years of operation, since this is enough time to conduct the study of the territory, define the demand and the coverage area and get to know the users and their biopsychosocial history. 

Two methods have been developed and, for both, the sample consisted of male users, aged eighteen years or more, assisted in the health services selected, from January to December 2011, through the review of their medical records. The choice of this period is justified to avoid duplication or loss of data, since 2011 was the last year in which there was only one information system active and implemented for years. The data collection was carried out between June and August 2013.

In the first method, a cross-sectional design was used to observe the relationship between the percentage of absences in the scheduled visits and the number of NCCD, estimated by the linear correlation coefficient of Pearson. 

158 medical records were randomly selected using the R software, version 2.11.0. We conducted the survey of secondary data of such users, including: age (years), marital status, occupation, education and absence percentage variables between the sessions scheduled in 2011 and the total of NCCD. These were grouped based on the International Classification of Diseases (ICD) ^(^ ^9^.

The second method, also a cross-sectional design, used the geoprocessing as a tool to observe the relationship between spatial distribution of NCCD and the spatial distribution of SV ^(^ ^8^. The sample was defined from the geographical location of each of the units, correlated to the map of SV ^(^ ^8^.

The SV is classified into eight groups: 1. Extremely low, 2- Very Low, 3- Low, 4- Medium (urban), 5 High (urban), 6- Very high (subnormal agglomerates), 7- High (rural) and not classified^8^. This classification is used because the municipality of the study belongs to the State of São Paulo, understanding that comparisons throughout the work would be more suitable to local conditions. 

Thus, 1250 medical records belonging to units distributed in the SV groups of 2 to 5 were selected, since these are the groups that were identified in the coverage areas^8^. The variables of this stage were: age (in years), marital status, race, occupation, SV group, full address and NCCD. This selection was random, done by the R *software* version 2.11.0. 

For the analysis the program *Geostatistics for the Environmental Sciences (GS+)* version 7.0, was used to generate semivariograms. It is noteworthy that the semivariogram is a measure of geological variability conditioned by distance, which shows the measure of spatial dependence between samples ^(^ ^10^. From the result of these semivariograms it was possible to determine the form of data interpolation.

After this procedure, using the ICD^9^, maps of the NCCD were generated, by kriging, which is defined as an estimate process of variable values distributed in space, from adjacent values considered as interdependent values by the semivariogram. Thus, for diseases that had spatial dependence kriging was used and for those who did not present spatial dependence, we used the inverse of the distance^10^. For this, we used the ArcGIS software, version 10.1.

With the maps created, the age range was superimposed on them to investigate the relationship between age and concentration of the NCCD. Finally, the classification of SV^8^ was superimposed on maps of the NCCD to verify the relationship between the classification of the SV and the concentration of the NCCD.

## Results

In the first method 158 medical records were analyzed, in which we studied the relationship between the user's age (years), marital status, occupation and education and percentile of absences with the number of diseases classified in ICD and number of NCCD.

Table 1 shows the data of the Spearman correlation between the age of the patient, marital status, occupation and education and percentile of absences with the number of ICD and number of NCCDs. It was observed that the larger the number of absences the fewer NCCD and the lower the amount of ICD. Furthermore, with increasing age, the amount of ICD and NCCD also increase. There was a higher amount of ICD and NCCD among men with a female partner.

**Table 1:** Relationship between the user age (in years), marital status, occupation and education and percentile of absences with the number of diseases from the ICD and the number of NCCDs. Botucatu, SP, 2013.

	Nº of diseases in the ICD*		Nº of NCCDs^†^	
AGE (Years)	r = 0,46; p < 0,001		r = 0,46; p < 0,001	
Marital Status				
Single (No female partner) (n=43)	1 (0 - 5)	p = 0,008	3 (0 - 9)	p = 0,012
Stable Union (With a female partner) (n=97)	2 (0-5)		4 (0 - 14)	
Occupation				
Working (n=32)	1 (0 - 4)	p = 0,277	2 (0 - 10)	p = 0,363
Not working (n=18)	1,5 (0 - 4)		3 (0 - 9)	
With at least High school complete				
No (n=97)	2 (0 - 5)	p = 0,142	4 (0 - 14)	p = 0,071
Yes (n=29)	1 (0 - 4)		2 (0 - 8)	
% de absences	r = -0,22; p = 0,005		r = -0,21; p = 0,006	

*International Classification of Diseases; †Chronic Non-communicable Diseases.

Next, the Spearman correlation was used between the percentile of absences with the diseases classified in the ICD and the number of NCCDs, corrected by age and stratified by marital status, occupation and level of education (Table 2). There was a relationship between the percentage of absences and illnesses from ICD in men with a female companion, the higher the percentage of absences, the lower the number of NCCDs and the number of diseases from ICD. Among those with less education (below high school), the higher the percentage of absences, lower the number of diseases from ICD and the lower the number of NCCDs

**Table 2:** Spearman correlation between the percentile of absences to the number of diseases from ICD and the number of NCCDs corrected by age and stratified by marital status, occupation and level of education. Botucatu, SP, 2013

	Nº of diseases in ICD*		Nº of NCCDs^†^	
Age (Years)	r = -0,13; p >0,05		r = -0,12; p >0,05	
Marital Status				
Single (No female partner) (n=43)	r = -0,02; p = 0,864		r = -0,01; p = 0,934	
Stable Union (With a female partner) (n=97)	r = -0,22; p = 0,025		r = -0,02; p = 0,029	
Occupation				
Working (n=32)	r = -0,18; p = 0,315		r = -0,14; p = 0,424	
Not working (n=18)	r = -0,44; p = 0,062		r = -0,38; p = 0,114	
With at least High school complete				
No (n=97)	r = -0,31; p = 0,002		r = -0,31; p = 0,002	
Yes (n=29)	r = -0,07 p = 0,711		r = -0,01; p = 0,932	

*International Classification of Diseases; †Chronic Non-communicable Diseases.

When observed the result on marital status, it was decided to check the factor which most interferes with the NCCD, whether it is marital status or age. According to the data collected, users who live in stable union are younger (Stable Union = 53 years (18-84) SV . Singles = 57 years (31-93), p = 0.008, Mann-Whitney test) (results represented as median (minimum-maximum)).

In addition, using the Spearman correlation between ages with the number of diseases listed in the ICD and the number of NCCDs, stratified by marital status, it was decided to verify the marital status in which the correlation between age and NCCDs is stronger. It can be concluded that it is stronger among singles (r = 0.58 among singles versus r = 0.28 among men in stable union, with p <0.01 for both). 

In the second method, the ICD diseases who had spatial dependence, i.e., those that were geostatistically significant, were neoplasms (cancer of the colon and prostate) and symptoms, signs and abnormal clinical and laboratory findings, not classified elsewhere (glucose intolerance), whose cases are listed in tables 3 and 4. The most likely probability to find men diagnosed with neoplasia was 55.21% to 69% in some regions, with different classifications for social vulnerability, as it can be seen in Table 3.

**Table 3:** Characteristics of patients with statistically significant results according to age and social vulnerability (VS) considering neoplasia in urban areas. Botucatu, SP, Brasil, 2013

User*	Age (years)	Types of Neoplasia	Classification concerning social vulnerability
VL55	60	Colon Cancer	2
CSE105	79	Prostate cancer	2
JI13	63	Prostate cancer	5
RJ9	84	Prostate cancer	2
SE12	53	Prostate cancer	5

*Codification of geostatistically significant cases.

**Table 4:** Characterization of patients with significant geostatistical results according to age and social vulnerability (VS) considering glucose intolerance, in the urban area. Botucatu, SP, Brasil, 2013

Patient*	Age (Years)	Classification concerning social vulnerability
CS16	57	2
CS76	No information	2
VL16	83	2
VL35	59	2
VL55	60	2
VL69	71	2
VL68	55	2
JP147	66	2
SL10	77	2
SL16	83	4
SL28	72	2
SL50	59	3
SL56	68	2
SL98	53	4
SL72	51	3
SL59	No information	4
SL23	55	2
VF35	61	3
VF36	48	2
RJ95	65	4
RJ141	No information	5

*Codification of geostatistically significant cases.

In Table 4 are the data regarding the diagnosis of glucose intolerance according to the age and social vulnerability, ranging from 63% to 79% in some regions.

From these results, it was decided to investigate, using the Pearson correlation between age (in years) and ICD neoplasms and ICD glucose intolerance, the likelihood of finding users with these NCCDs that were associated with age. It is noticed that, although statistically significant (p <0.05), there is a very low correlation between age and neoplasia (r = 0.071) and age and glucose intolerance (r = 0.103). It was also noted that as far as the SV classification is concerned, most users are in the classification 2, considered very low^8^.

## Discussion

In this study, it was observed that for men, the younger the age, the lower the number of chronic diseases and the higher is the number of absences in scheduled appointments. The older the subjects, the greater is the number of NCCDs and the lower the number of absences in the scheduled visits, showing that between absences and chronic diseases there is an inverse ratio. 

Comparing to literature data, regardless of sex, other studies found similar data, such as a study that, from a total of 22 people, 64% of assiduous patients in scheduled medical consultations were over 60, 82% of defaulters had less than 60^11^ ^)^ and the proportion of absentees decreases with the increasing age of subjects^12^. This fact is possibly due to a number of NCCDs already installed in the elderly, which would require closer monitoring. Therefore, it was observed that elderly people with poor health there is less absent in routine medical appointments, probably, because they have greater health needs compared to younger seniors, since comorbidities increase with age^13^ or due to the accumulation of experience that leads the user to take the opportunity of an appointment to identify early health problems ^(^ ^14^. 

Furthermore, it was found that among those with lower education (below high school), the greater the percentage of absences, the fewer the number of diseases mentioned in the ICD and the smaller is the number of NCCDs, that is, those men who had fewer NCCDs associated and had lower education were the most absent.

The lack of preventive care, including the inadequate monitoring can lead to complications and often the need for hospitalization. A study carried out in the Brazilian city of Maringa, in the period between 2000 and 2011, found that the causes of hospitalization among men occurred mainly due to mental disorders, injuries and respiratory diseases and that the hospitalization in the second triennial period of the study, had an overall average higher than among women, probably because it there is a delay in seeking care, worsening health status ^15^. 

Regarding the geoprocessing, the spatial dependence was only obtained in the urban area of the following comorbidities: neoplasms and glucose intolerance. A study carried out in the United States showed an estimate of the new cases of cancer that affect men. Among them, the greatest impacts are in prostate, lung, bladder and colon cancer ^16^ ^))^. This research converges with the estimate of the National Cancer Institute (INCA - In Portuguese) which cites these organs besides the skin and esophagus ^(^ ^17^.

These data corroborate with the findings in the present study that showed five cases of cancer, geostatiscally significant, one being colon cancer and four prostate cancer. Risk factors associated with colon cancer range from the interaction of environmental factors such as a red meat diet, dairy protein, coffee, among others ^(^ ^18^, to the presence of endogenous factors such as family history of colorectal cancer, genetic predisposition to developing chronic bowel disease and age, the incidence and mortality increase with age ^(^ ^17^. 

The case of colon cancer found in this study is probably associated to the age (60 years), since that the subject lived in a region with the lowest SV found in the municipality (VS 2). Moreover, he had as chronic comorbidities hypertension and glucose intolerance, indicating, perhaps, poor diet or genetic causes. However, it was not possible to affirm such issues for the data found in the medical records were punctually related to the diseases he had with no information regarding his diet or family history.

The other significant type of cancer in this study was the prostate that stands out as a major problem of public health worldwide, with a progressive increase in its incidence in several countries ^(^ ^19^. Related to personal issues, the main risk factors described for the development of prostate cancer are: age ^(^ ^16^, ethnicity ^(^ ^20^ and family predisposition ^(^ ^21^. In addition, first-degree relatives of patients with prostate cancer relatives are at increased risk of developing this cancer when compared to men in the general population ^(^ ^22^.

This study found four cases of prostate cancer. The subjects with this disease had 53, 63, 79 and 84 years. Concerning age, this research converges with the incidence of the disease reported by other authors, showing that this type of cancer increases with age, starting with a probability of 0.3% after 49 years, rising to 10.9% in the range age of 70 years or more^16^.

The subjects of 79 and 84 years, living in public sites classified as SV 2. As for the ones with 53 and 63, in regions classified as SV 5 (Very High), that is, living in socially vulnerable areas. Added to this, the subject with 63 was a retired gardener, indicating that he had a low socioeconomic status. The study points out that a significant part of neoplasia is attributed to environmental influences, particularly those related to lifestyle. Thus, factors related to quality of life influence significantly in the occurrence of prostate cancer ^(^ ^23^.

In relation to food issues and family history, it was not possible to make any statement because these data were not included in the notes of the charts. The second significant harm found in this study, was glucose intolerance. There were 21 geostatiscally significant cases of glucose intolerance in the urban area of the municipality. Of these, 62% lived in the region with the classification of SV Very Low, 19% Medium, 14% low and 5% High. 

The patients who had information on date of birth and suffered from impaired fasting glucose (IFG), 100% were older than 40 years. These findings may reflect the impact of environmental factors and lifestyle, but age is the factor that determines the greatest risk to changes in glucose homeostasis ^(^ ^21^, corroborating this work. Studies show that the incidence and prevalence of type 2 diabetes sharply increases with increasing age, especially after 40 years ^(^ ^24^.

## Conclusion

We sought to evaluate in male patients, the association between the percentage of absences in the scheduled visits and the number of NCCDs and to investigate the relationship between spatial distribution of NCCDs and the spatial distribution of SV .

It was observed that the older the men, the greater the number of NCCDs installed and the less they miss scheduled appointments. It was also noted that a higher amount of diseases listed in the ICD and NCCDs was found among men with a female partner and that the level of education interferes in the percentage of absences, being higher in individuals with low education. Thus, it was noted that there is an inverse relationship, confirming one of the hypothesis, between the number of absences and the number of diseases listed in the ICD and NCCDs present.

Regarding the use of geoprocessing, we obtained a significant number of cases of glucose intolerance in urban areas, the majority classified as SV 2, perceived as very low. It is noteworthy that it was possible to relate the spatial distribution of NCCDs with the SV 2, however, the same did not happen with the relationship of NCCDs and higher levels of SV , covering in part one of the hypothesis of the study. 

However, it is emphasized that the use of GIS in health care has been facilitated by universal access to epidemiological databases and the provision of cartographic tools and computerized statistics. These advances enable the rapid production of thematic maps that can contribute to the formulation of hypotheses about the spatial distribution of health problems and its association with socioeconomic indicator data, providing the intersection of epidemiological indicators with each other, facilitating the inclusion of intersectoral data and the relationships between living conditions, health and disease. 

We emphasize the paucity of researches similar to this one, because most of them characterize the population studied, making a descriptive quantitative approach without the correlation between health status, personal and socioeconomic characteristics and spatial distribution.

The use of geoprocessing enabled the creation of thematic maps on NCCDs and SV, thereby contributing to the improvement of the understanding of the association between spatial distribution of health problems and socioeconomic indicators data. In this way the study tried to help in the identification of epidemiological patterns, to assist in the control and prediction of these diseases.

Looking at these results, we can see how much they can contribute to the development of the nursing work in the sense of planning actions that interfere directly in the active search for absentees, as well as in mapping the areas with different social vulnerability ratings and in this way, finding tools that assist in promoting the health of a clientele that is finding it difficult to access health services. 

It should be noted that limitations occurred, such as difficulties in the analysis of the records, because of the few number of records or the lack of them on the part of health professionals, as well as the difficulty in mapping the regions of the city, especially the countryside.
